# Risk Assessment of Chlorothalonil as a Probable Human Carcinogen on Selected Vegetables in an Eastern China Province

**DOI:** 10.3389/fpubh.2022.917269

**Published:** 2022-07-06

**Authors:** Chen-Xi Sun, Bing Liu, Wen-Bo Wang, Xue-Xia Yuan, Yuan-Juan Wu

**Affiliations:** ^1^Shandong Academy of Agricultural Sciences, Shandong Provincial Key Laboratory of Test Technology on Food Quality and Safety, Jinan, China; ^2^Resources and Environment Innovation Institute, School of Municipal and Environmental Engineering, Shandong Jianzhu University, Jinan, China

**Keywords:** chlorothalonil, carcinogenic risk assessment, pesticide residue, public health, Monte-Carlo simulation

## Abstract

**Objectives:**

This study aimed to provide an assessment of chlorothalonil's possible carcinogenic risk posed to the public. In combination and comparison with the non-carcinogenic risk, the results hopefully could provide useful insights, early warning, and references for policy formulation.

**Methods:**

This study firstly investigated the occurrence of chlorothalonil on selected key vegetables for different scenarios, and then conducted an exposure assessment with officially published data. Lastly, both the non-carcinogenic and carcinogenic risk of chlorothalonil were calculated by using Monte-Carlo simulation.

**Results:**

Even though mean non-carcinogenic risks of chlorothalonil for all scenarios were below threshold value, the mean carcinogenic risks for maximum-risk scenario and most-likely risk scenario were mostly above threshold value. High probabilities of exceedance of threshold value existed for carcinogenic risk under all scenarios.

**Conclusion:**

Potential threat to public health existed for conventionally ‘safe' pesticide if considering the possible carcinogenicity. Extra caution should be taken and the potential carcinogenic effects should be included into consideration for better protection of public health during the policy formulation process.

## Introduction

Chlorothalonil(2,4,5,6-tetrachloroisophthalonitrile, CAS 1897-45-6) is a broad-spectrum fungicide that has been used widely across the world to protect vegetables, fruits, turf, and ornamental plants (1). Since its invention in the 1960s, it has been considered to have low toxicity (2). However, new evidence has been emerging and its carcinogenicity has gradually been proved on animals (3, 4). As a result, the International Agency for Research on Cancer (IARC) and the United States Environmental Protection Agency (USEPA) have both declared it a probable human carcinogen (5, 6).

As a response, a number of countries have conducted a re-evaluation of chlorothalonil's safe use and adjusted their management policies. For example, New Zealand issued red alert to ban chlorothalonil use outside of the workplace in 2017 (7). Pest Management Regulatory Agency (PMRA) of Canada canceled the uses of chlorothalonil on greenhouse cut flowers, greenhouse pachysandra, and field-grown roses (for cut flowers) in 2018 (8). European Union terminated chlorothalonil renewal of approval in 2019 followed by a complete ban in 2020 (9). Even though these and other countries have all tightened their chlorothalonil management, it is clear that disagreement still exists on how exactly chlorothalonil should be restricted for use.

Dietary intake is one of the most important exposure pathways of chlorothalonil to the public (10). However, most of the present chlorothalonil management guidelines or policies, such as ADI (acceptable daily intake), ARfD (acute reference dose), or MRL (maximum residue level) on food were mainly derived based on chlorothalonil's chronic toxicity and acute toxicity without much consideration of its potential human carcinogenicity. This might result in an underestimation of the risk and lead to insufficient protection of the public. Therefore, this study aimed to conduct a dietary risk assessment of chlorothalonil taking into consideration both its non-carcinogenicity and potential carcinogenicity. Hopefully, it could provide useful insights, early warning, and references for improving chlorothalonil management for public health protection.

## Methods

### Overall Design

China is one of the top consumer countries of chlorothalonil (11), where the chemical is primarily used for vegetables and fruits, etc. As one study cannot cover all exposure pathways, this study selected several important vegetables to detect chlorothalonil's residue concentration, estimate its exposure dose, and calculate its health risks based on different scenarios.

The three scenarios were set in this study, (1) maximum possible risk posed to the public under current management conditions, which was indicated by vegetables collected with minimum pre-harvest interval (3 days) from the greenhouses (12); (2) most-likely risk posed to the public, which was indicated by vegetables collected from the markets; (3) control conditions, which was indicated by assuming chlorothalonil residue at MRL.

The 4 types of vegetables, tomato, cucumber, celery, and pepper, were collected for each scenario as these were vegetables that raised most concern for public health during routine monitoring projects. After collection, the samples were prepared and analyzed with gas chromatograph (GC). Occurrence of chlorothalonil on these samples was expressed as probability distribution function, and risks were calculated by using Monte-Carlo simulation.

### Sample Collection and Preparation

Vegetable samples were collected from 17 counties in Shandong Province of China during April and May 2020. For Scenario 1, 174 samples of each vegetable were collected from Yaoqiang Vegetable Base, Jinan, Shandong. The 3 days after chlorothalonil application, approximately 1,000 g of each sample was collected from top, middle, and bottom part of the same plant and mixed thoroughly before sealed in sterile plastic bags. A total of 696 samples were collected. For Scenario 2, the same 4 types of vegetables were collected from supermarkets, farmers' markets and grocery stores in a random pattern. 1,000 g of each sample was collected. A total of 1,032 samples were collected with 258 for each type of vegetables. No samples were collected for control conditions. The collected samples were transferred to lab in ice boxes and stored at −20°C for further analysis. Before GC analysis, each sample was shredded, grinded, and mixed thoroughly for chlorothalonil extraction, purification, and detection.

### Chlorothalonil Detection

Chlorothalonil residue on vegetable samples was detected using a GC method. In Brief, chlorothalonil was firstly extracted with acetonitrile (HPLC grade, Fisher Scientific,) from the vegetable samples. The 25.0 g of sample was mixed with 100.0 ml acetonitrile and then homogenized (IKA T18 basic rapid) for 2 min before filtration. The filtrate was mixed with 5.0 g NaCl (Analytical grade, Sinopharm Chemical Reagent) and vortexed for 1 min. Let the mixture stand at room temperature for 30 min for separation. The supernatant was recovered for chlorothalonil purification. Pass the supernatant through a solid-phase extraction column (Bond Elut, Agilent Technologies), and reduce the volume to below 2.0 ml with a rotary evaporator (Heidolph Laborita 4,000). The concentrate was adjusted to 2.0 ml with hexane (HPLC grade, Fisher Scientific), and then analyzed by a GC (Agilent 6890N) equipped with an electron-capture device (ECD) and a DB-5MS GC column (30 m × 0.25 mm × 0.25 μm). The Nitrogen (99.999%, Deyang Special Gas) was used as the carrier gas and maintained at a constant flow rate of 1 ml min^−1^. A sample volume of 5.0 μl was injected. The inlet and detector temperatures were held at 200 and 320°C, respectively. The column temperature was programed as follows: initial temperature, 150°C, held for 2 min, increased to 270 at 6°C min^−1^, held at 270°C for 8 min. The limit of quantification (LOQ) of the method was 0.01 mg kg^−1^.

### Probability Distribution

In order to conduct a risk assessment as accurately as possible, a proper probability distribution for chlorothalonil occurrence data was necessary. The residue data for Scenario 1 and Scenario 2 were both fitted using Minitab as described previously (13). The Individual Distribution Identification function was used to estimate data distribution for five probability functions (normal, lognormal, Weibull, exponential, and logistics) of the data without non-detects. The censqq function was used for the interval censored data as described previously (13). Anderson–Darling test (parameter AD) and goodness-of-fit test (parameter *p*) were used to evaluate the fitness of each distribution. Only distribution with a *p*-value > 0.05 was considered a proper fit, and the distribution with smallest AD and highest *p*-values were selected.

It was possible that no fitness of distribution was observed for the original datasets. Under such circumstances, a Johnson transformation was used to transform the target dataset into normal distribution. AD and *p*-values were also used as the selection criteria.

### Risk Assessment

The exposure dose of chlorothalonil was calculated as the production of residue concentration and vegetable consumption. The probabilistic non-carcinogenic risk and carcinogenic risk were both calculated using Monte-Carlo simulation with Oracle Crystal Ball (version 11.1.2.2, Oracle, Inc., USA) (14, 15).

Vegetable consumption data for “North China” from “*Exposure Factors Handbook of Chinese Population”* were used (16). Vegetable consumption was divided into two groups, being dark-colored vegetables and light-colored vegetables. In this study, tomato, celery, and pepper were categorized as dark-colored vegetables, and cucumber was categorized as light-colored vegetables. The original vegetable consumption data were classified according to gender (Female/male), age (2–5, 6–17, 18–29, 30–44, 45–59, 60–69, 70, and above) and habitation (urban/rural). In order to reduce the unnecessary complexity of the assessment, a paired *t*-test was used to evaluate the difference among different groups. Results showed that significant difference existed between males and females (*p* = 0.003), adults, and children (*p* = 0.023), but no significant differences (*p* = 0.121) were observed between people living in urban and rural areas. The vegetable consumption data were then simplified accordingly (Table 1).

**Table 1:** Vegetable consumption (g/d) (East China, China, 2013).

**Veg name**	**Mean**	**Male-adult**	**Female-adult**	**Children(2–5)**
Dark-colored veg	90.8	94.32	87.8	42.65
Light-colored veg	185.4	196	179.28	82.18

Body weight information for “North China” from “*Exposure Factors Handbook of Chinese Population”* was used to estimate the per body weight chlorothalonil intake (17). To be consistent with the vegetable consumption data, the body weight data were recombined, calculated, and categorized into three groups (male-adults, female-adults, and children). All body weight data follow normal distribution (Table 2). The mean, five percentile, and 95 percentile values were entered into Crystal Ball software to define the probability distribution.

**Table 2:** Body weight distribution (kg) (East China, China, 2013).

	**Mean**	**P5**	**P25**	**P50**	**P75**	**P95**
Male-adults	69.7	52.9	61.5	68.6	76.3	90.6
Female-adults	61.5	46.8	54.6	60.8	67.3	78.8
Children(1–5)	33.0	12.0	19.0	30.0	44.0	62.0

The non-carcinogenic chronic risk of chlorothalonil was determined based on the ‘Targeted Hazard Quotient (THQ)' method (14, 18, 19) and expressed as Eq. (1). The per bodyweight daily intake of chlorothalonil was compared with the ADI value, and a value > 1 indicated unacceptable risk (18, 20).


(1) $$\begin{array}{r} R_{n}=\frac{C_{ch}\times Q_{veg}}{bw\times ADI}\ldots \ldots \ldots \ldots \ldots \ldots \ldots . \end{array}$$

Where *R*_*n*_ is the non-carcinogenic risk of chlorothalonil, *C*_*ch*_ is the chlorothalonil residue concentration detected on the samples (*mg/kg*), *Q*_*veg*_ is the vegetable consumption per day (*kg*), *bw* is body weight (*kg*), *ADI* is the acceptable daily intake (*mg/(kg*·*bw)*). In risk calculation, the chlorothalonil residue concentration *C*_*ch*_ was expressed as the function of the fitted probability distribution, or the inverse function of the fitted distribution of Johnson transformation; *Q*_*veg*_ was defined as either the dark-colored or the light-colored vegetable consumption in Table 1.

The carcinogenic risk of chlorothalonil was calculated based on the linearized multi-stage model (21) and expressed as Eq. (2). The oral slope factor of chlorothalonil q^*^ was adopted from Raman (10) as 0.00,766 mg/(kg·d). The risk was compared with a threshold cancer risk level of 10^−4^, which was used by USEPA as a cancer risk reference point (22).


(2) $$\begin{array}{r} R_{c}=1-exp\left[-\left(q^{{}^{*}}\times C_{ch}\times Q_{veg}\right)\right]\ldots \ldots . \end{array}$$

Where *R*_*c*_ is the carcinogenic risk.

## Results

### Occurrence of Chlorothalonil Residue on Selected Vegetables

For Scenario 1, chlorothalonil was detected on all vegetable samples. The mean residue levels on tomato, cucumber, celery, and pepper were 0.58, 0.18, 4.65, and 0.10 mg/kg, respectively (Supplementary File 1). For Scenario 2, the positive rate was significantly lower than that of Scenario 1, 10.08, 13.33, 6.29, and 7.78% of tomato, cucumber, celery, and pepper samples were detected positive, respectively. The mean residue levels of the positive samples on tomato, cucumber, celery, and pepper were 0.53, 1.55, 4.42, and 1.15 mg/kg, respectively. With the non-detects, the mean residue levels on tomato, cucumber, celery, and pepper collected from the market were 0.02, 0.10, 0.44, and 0.19 mg/kg, respectively (Supplementary File 1).

Most of the residue concentration data did not fit into any of the pre-defined probability distribution functions, but they could fit well into normal distribution after the Johnson transformation (Table 3).

**Table 3:** Probability distribution of chlorothalonil on different samples.

**Sample names**	**Distribution function after transformation**	**Transformation equation**	**Fitness parameters**
Tomato${}_{\text{G}}^{\text{a}}$	N(0.0188, 0.981^2^)	*y* = 2.173 + 0.891 × Ln((X + 0.022)/(5.077– X))	AD = 0.133, *P* = 0.98
Cucumber_G_	N(0.01264, 0.996^2^)	*y* = 3.591 + 0.948 × Ln((X + 0.012)/ (5.423–X))	AD = 0.17, *P* = 0.933
Celery_G_	N(0.00471, 1.101^2^)	y=-0.687 + 0.864 × Ln X + 0.107)	AD = 0.31, *P* = 0.553
Pepper_G_	N(0.0022, 1.018^2^)	*y* = 1.793 + 0.755 × Ln((X−0.004)/(0.694–X))	AD = 0.265, *P* = 0.692
Tomato${}_{\text{M}}^{\text{b}}$	*N*(0.0141, 1.153^2^)	*y* = −1.508 + 0.817 × Asinh((X−0.00,514)/0.00,195)	AD = 0.316, *P* = 0.521
Cucumber_M_	*N*(0.00,417, 0.908^2^)	y=1.703 + 0.516 × Ln((X + 0.000,810)/(1.173–X))	AD = 0.246, *P* = 0.71
Celery_M_	*N*(0.006, 1.168^2^)	*y* = −0.712 + 0.254 × Asinh((X−0.0161)/0.000,426)	AD = 0.236, *P* = 0.715
Pepper_M_	*N*(0.191, 0.142^2^)^c^	/	AD = 0.254, *P* = 0.578

*^a^, the subscript G indicated samples collected for Scenario 1 from the green houses*;

*^b^, the subscript M indicated samples collected for Scenario 2 from the market*;

*^c^, residue level data of pepper collected for Scenario 2 from market follows normal distribution, no transformation was needed*.

### Non-carcinogenic Risk of Chlorothalonil

**Figure 1:**
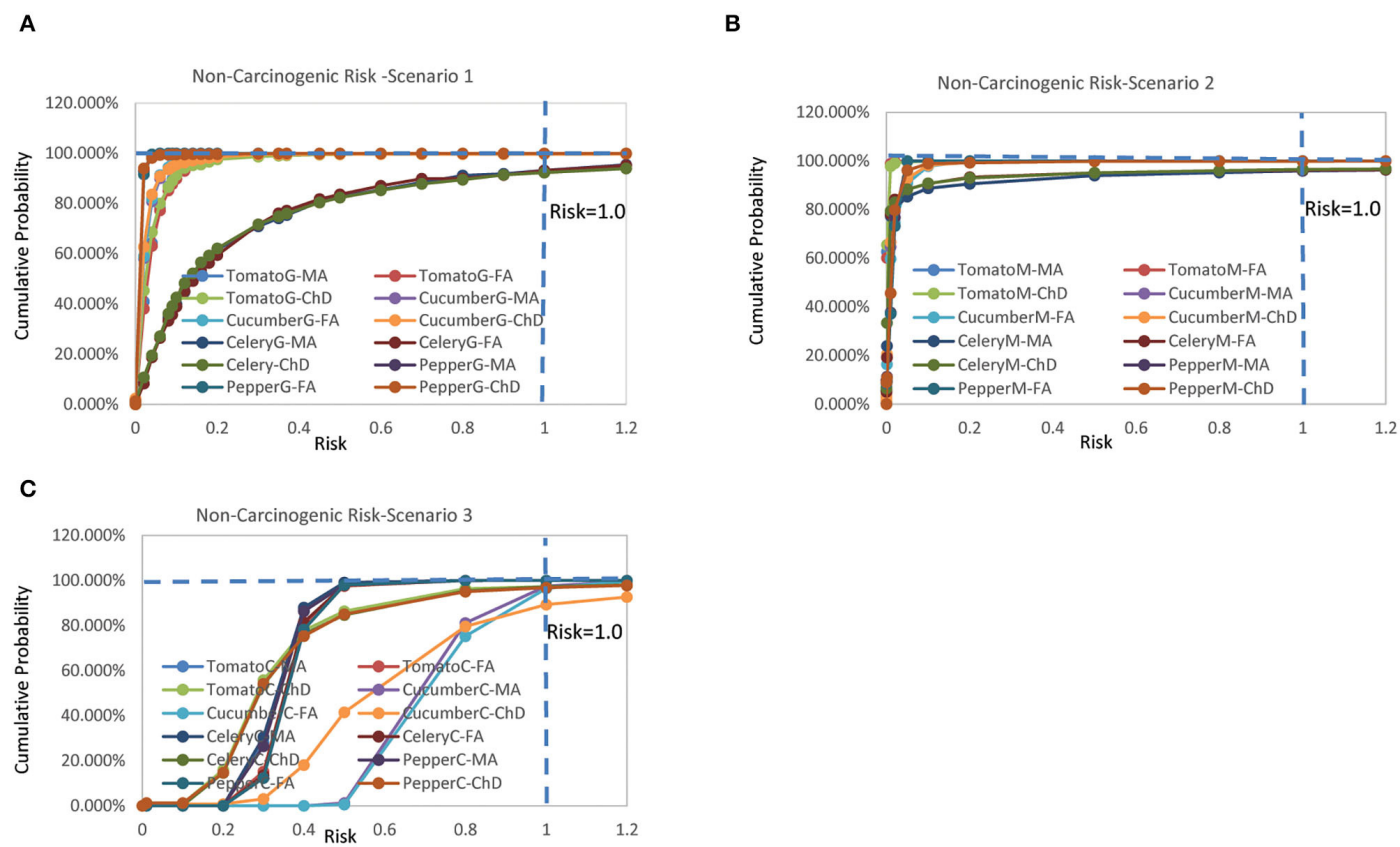
Cumulative probability of non-carcinogenic risks for **(A)** scenario 1, **(B)** scenario 2, and **(C)** scenario 3 (Jinan, China, 2021).

The non-carcinogenic chronic risks for the three scenarios rank as Scenario 3 > Scenario 1 > Scenario 2 (Figure 1). It was found that the risks for different population groups of the same type of vegetable exhibited similar patterns with similar mean risk value. The inter-species difference between different vegetables was very significant, and positively related to the residue concentration. The estimated mean non-carcinogenic risks extended a wide range from 0.00,073 to 0.729 (Supplementary File 2).

For Scenario 1, the estimated mean risks were all below one, and ranked as celery > tomato > cucumber > pepper, with female adult > male adult > children for all vegetables. There was no probability of risk exceeding the threshold value of 1 for tomato, cucumber, and pepper for all population groups. The probabilities of risk exceeding one for celery were 6.83% (female adult), 6.75% (male adult), and 7.66% (children).

For Scenario 2, the estimated mean risks were all below one, and ranked as pepper > cucumber > celery > tomato, with female adult > male adult > children for all vegetables. There was no probability of risk exceeding the threshold value of one for tomato, cucumber, and pepper for all population groups. The probabilities of risk exceeding 1 for celery were 3.74% (female adult), 4.08% (male adult), and 3.51% (children).

For Scenario 3, the estimated mean risks were all below one, and ranked as cucumber > tomato = celery = pepper, with female adult > male adult > children for all vegetables. It was found the probabilities of risk exceeding one were 3.49% (female adult), 2.49% (male adult), and 10.76% (children) for cucumber, and 2.78% (tomato, children), 3.18% (pepper, children), and 2.8% (celery, children).

### Carcinogenic Risk

**Figure 2:**
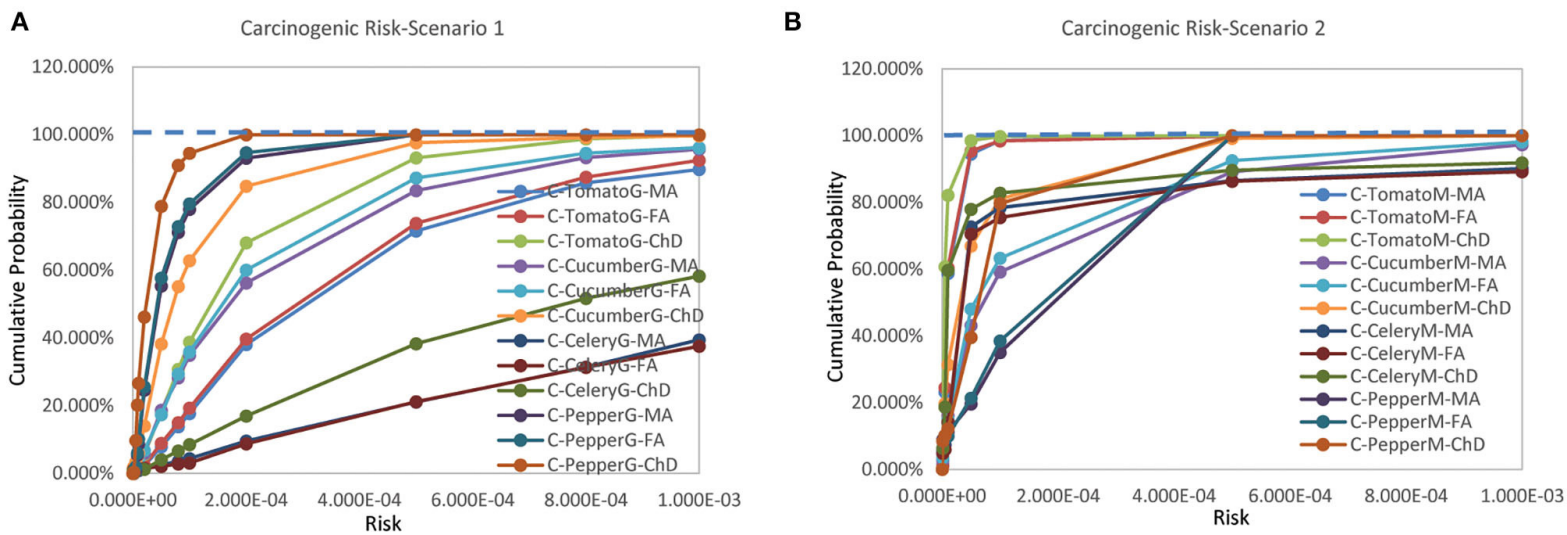
Cumulative probability of carcinogenic risks for **(A)** scenario 1 and **(B)** scenario 2 (Jinan, China, 2021).

The carcinogenic risks for the three scenarios rank as Scenario 3 > Scenario 1 > Scenario 2 (Figure 2). Compared with the non-carcinogenic risks, carcinogenic risks of different vegetables from different population groups exhibited greater variation. The estimated mean carcinogenic risks extended a wide range from 3.653 × 10^−6^ to 7.479 × 10^−3^ (Supplementary File 3).

For Scenario 1, the estimated mean risks for pepper of all population groups and celery for female adult were below 10^−4^. The estimated mean risks for tomato and cucumber of all population groups as well as celery of male adults and children were above 10^−4^. Probabilities exceeding 10^−4^ existed for all vegetables of all population groups (Supplementary File 4).

For Scenario 2, the estimated mean risks for tomato, cucumber, and celery of all population groups as well as pepper for children were below 10^−4^. The estimated mean risks for pepper of male and female adults were above 10^−4^. Probabilities exceeding 10^−4^ existed for all vegetables of all population groups, but the likelihood was significantly lower than that of scenario 1 (Supplementary File 4).

For Scenario 3, the estimated mean risks for all vegetables of all population groups were > 10^−4^. As no distribution functions were available for Scenario 3, no probabilities exceeding 10^−4^ were calculated.

## Discussion

### Uncertainty of Risk

The non-carcinogenic and carcinogenic risks of chlorothalonil on selected vegetables (tomato, cucumber, celery, and pepper) were calculated in this study. The non-carcinogenic risk was dependent on the residue concentration, vegetable consumption, body weight, and ADI. Among the four parameters, residue concentration contributed over 80% of the risk variation. Therefore, it was into probability distribution function to account for the variation of the data. Among the rest parameters, vegetable consumption, and ADI values could be the two parameters that introduce most error in the results. Firstly, vegetable consumption data were gathered from the “*Exposure Factors Handbook of Chinese Population”*, which was published in 2013. Almost a decade has passed, the dietary pattern of the country may have evolved (23) and errors could be induced as a result. Meanwhile, vegetable consumption data was simply divided into two categories (“dark-colored” vegetable and “light-colored” vegetable), and no distribution function was used. In the actual exposure and risk calculation process, the consumption of each of the four vegetables was assumed to be equal to either “dark-colored” vegetable or “light-colored” vegetable, which was an over-simplification. The actual vegetable consumption may be significantly lower than that of used in the assessment. As a result, the risk may be systematically overestimated.

Secondly, the ADI adopted by this study was 0.02 mg/(kg·bw) (24), which was at the upper limit of the FAO's (food and agriculture organization) recommendations of 0–0.02 mg/(kg·bw) (25). Even though some countries, such as South Korea, may have been using a higher ADI [0.03 mg/(kg·bw)] (26), the current calculation might still result in an underestimation of the actual risk.

Lastly, this study did not take into consideration the processing factor (PF) of chlorothalonil and assumed all the vegetables consumed unwashed and raw. However, the previous studies found that the PF of chlorothalonil for tomatoes was 0.01–0.10 for thermal treatment, 0.25 for mechanical treatment, and 0.51 for chlorinated water washing (27). As a result, the actual dietary intake and associated risk could be reduced by up to 100 times.

### Risk Levels

The chlorothalonil residue concentration on market vegetables and greenhouse vegetables were detected in this study. For the market vegetables, 10.08, 13.33, 6.29, and 7.78% of tomato, cucumber, celery, and pepper were detected positive, respectively. Compared with similar studies, the positive rate of chlorothalonil was significantly lower than that in wax (49.2%) and pollen (52.9%) in the USA (28), and lower than that in pepper (25.2%) from Seoul (26).

The mean chlorothalonil residue concentrations on tomato, cucumber, celery, and pepper collected from the market were 0.02, 0.10, 0.44, and 0.19 mg/kg, respectively, which were all below the national MRL (5 mg/kg). These values were comparable with results from other parts of the world. For example, studies found that chlorothalonil residue concentration ranged from 0.03 to 0.50 mg/kg for Poland (27), 1–4.3 μg/kg in fruits of peach-nectarine cultivars in Greece (29), 0.045–31.039 mg/kg in Seoul (26), 0.43 mg/kg on strawberries and 15.435 mg/kg on lettuce in Romania (30). The mean residue concentrations on tomato, cucumber, celery, and pepper collected from greenhouse were 0.58, 0.18, 4.65, and 0.10 mg/kg, respectively. Similar study conducted in Egypt found chlorothalonil residue level was 0.79 mg/kg on tomato and 1.97 mg/kg on pepper (31). Overall, chlorothalonil residue levels detected in this study were of similar magnitude to the rest of the world.

Very different conclusions could be drawn from the non-carcinogenic and carcinogenic risk assessment. For the non-carcinogenic risk, the most-likely risk and maximum risk were 0.0,136 and 0.151, respectively, which were both significantly lower than the threshold value of one. Similar studies showed that mean chlorothalonil risk was 0.540 on unprocessed tomatoes in Poland (27), 3.09 on vegetables with 95% confidence interval of 2.54 to 3.91 in Seoul (26), and 0.368 for WHO cluster diet B population in Romania (30). Compared with the above-mentioned studies, the non-carcinogenic risk found in this study was mostly lower. For the possible carcinogenic effects, high probabilities existed for exceedance of the threshold value of 10^−4^. Among different vegetable and population groups, the maximum “most-likely” carcinogenic risk was approximately 40% higher than the threshold value, and the “maximum” carcinogenic risk was even 15 times higher.

However, it should be noted that chlorothalonil is classified as a probable human carcinogen up to date, and no confirmed evidence has been found yet between chlorothalonil application and human cancer incidences (32, 33). Therefore, the application of the carcinogenic risk assessment result during the policy formulation process should be carefully evaluated with economic and social considerations.

### Chlorothalonil Management

Maximum residue level is an important tool for pesticide management. Currently, the Chinese “National food safety standard-Maximum residue limits for pesticides in food” (24) specified chlorothalonil MRL on around 30 types of vegetables. Among these vegetables, MRL on potatoes was the lowest (0.2 mg/kg), and MRL on onions was the highest (10 mg/kg). More than half of these vegetables had a MRL of 5 mg/kg. Compared with other countries where 2 mg/kg for cabbage in Japan, 5 mg/kg in the United States and South Korea (34), 0.01 mg/kg in EU (35), the Chinese chlorothalonil MRL was not very strict. Especially, when considering the possible carcinogenic risk from Scenario 1 and Scenario 3, the present MRL might not be able to provide adequate protection to public health. Therefore, the possibility of a stricter chlorothalonil MRL could be considered.

A comparison between results from Scenario 1 and Scenario 2 indicated the importance of a proper pre-harvest interval. According to the pesticide registration information from *China Pesticide Information Network*, the minimum pre-harvest interval for chlorothalonil used on vegetables and fruits was 3 days, but 7 days was more commonly used for most vegetables (12). Compared with other countries, such as EU (45 days) and Canada (14 days), this value was quite short. Therefore, it could be a reasonable option to increase the minimum pre-harvest interval for a better protection of public health.

In conclusion, even though chlorothalonil has long been considered a ‘safe' fungicide with low toxicity, it might still pose a significant risk to the public when considering its possible carcinogenicity. Since its carcinogenic effects on human health cannot be completely ruled out, it might be necessary to include it into the policy formulation process, and implement a reasonably lower MRL and longer pre-harvest interval.

## Data Availability Statement

The original contributions presented in the study are included in the article/Supplementary Material, further inquiries can be directed to the corresponding author/s.

## Author Contributions

C-XS collected exposure data, constructed the model, performed the assessment, and prepared the first draft. BL designed and instructed the experiment. W-BW conducted the experiment and collected the data. X-XY reviewed and edited the draft. Y-JW organized the study. All authors contributed to the article and approved the submitted version.

## Funding

The authors declare that this study received funding from National Special Project for Quality Risk Assessment of Agricultural Products (GJFP2019005), Natural Science Foundation of Shandong province (ZR2020ME236), and National key Research and Development Program (2022YFE0105800). The funder was not involved in the study design, collection, analysis, interpretation of data, the writing of this article, or the decision to submit it for publication.

## Conflict of Interest

The authors declare that the research was conducted in the absence of any commercial or financial relationships that could be construed as a potential conflict of interest.

## Publisher's Note

All claims expressed in this article are solely those of the authors and do not necessarily represent those of their affiliated organizations, or those of the publisher, the editors and the reviewers. Any product that may be evaluated in this article, or claim that may be made by its manufacturer, is not guaranteed or endorsed by the publisher.

## References

[1] Wu X Yin Y Wang S Yu Y. Accumulation of chlorothalonil and its metabolite, 4-hydroxychlorothalonil, in soil after repeated applications and its effects on soil microbial activities under greenhouse conditions. Environ Sci Pollut Res Int. (2014) 21:3452-9. 10.1007/s11356-013-2318-1 24243264

[2] Costa LG Aschner M. Toxicology of Pesticides. Reference Module in Biomedical Sciences. Elsevier (2014). 10.1016/B978-0-12-801238-3.00208-7

[3] Castro MS Penha LCC Torres TA Jorge MB Carvalho-Costa LF Fillmann G. Genotoxic and mutagenic effects of chlorothalonil on the estuarine fish Micropogonias furnieri (Desmarest, 1823). Environ Sci Pollut Res. (2022) 29:23504–11. 10.1007/s11356-021-17328-2 34807392

[4] Van Scoy AR Tjeerdema RS. Environmental fate and toxicology of chlorothalonil. Rev Environ Contam Toxicol. (2014) 232:89–105. 10.1007/978-3-319-06746-9_4 24984836

[5] USEPA. R.E.D. facts chlorothalonil. Washington, DC: U.S. Environmental Protection Agency (1999).

[6] IARC. Some chemicals that cause tumors of the kidney or urinary bladder in rodents and some other substances. ARC Monogr Eval Carcinog Risks Hum. (1999) 73:183–93.

[7] NZEPA. Chlorothalonil use banned outside of the workplace. (2017). Available online at: https://www.epa.govt.nz/news-and-alerts/alerts/vtas.

[8] HCsPMRA. Re-evaluation Decision RVD2018-11, Chlorothalonil and Its Associated End-use Products for Agricultural and Turf Uses. (2018).

[9] EU. Commission Implementing Regulation (EU) 2019/677. (2019).

[10] Raman P. Chlorothalonil. In: Wexler P, editor. Encyclopedia of Toxicology. 3rd ed. Oxford: Academic Press (2014). p. 919–22.

[11] Wang C Lin J Xie T Zou H Shen Y. Determination of residual chlorothalonil in textiles. J Mat Sci. (2020) 8:106–14. 10.4236/msce.2020.84009

[12] China Pesticide Information Network. Industrial Database. (2021). Available online at: http://www.chinapesticide.org.cn/hysj/index.jhtml.

[13] Vergara GGRV Rose JB Gin KYH. Risk assessment of noroviruses and human adenoviruses in recreational surface waters. Water Res. (2016) 103:276–82. 10.1016/j.watres.2016.07.048 27472908

[14] Adegbola IP Aborisade BA Adetutu A. Health risk assessment and heavy metal accumulation in fish species (*Clarias gariepinus* and *Sarotherodon melanotheron*) from industrially polluted Ogun and Eleyele Rivers, Nigeria. Toxicol Rep. (2021) 8:1445–60. 10.1016/j.toxrep.2021.07.007 34401354 PMC8349904

[15] Fan B Wang X Xie Z Li J Gao X Cui L. Aquatic life criteria and human health ambient water quality criteria derivations and probabilistic risk assessments of 7 benzenes in China. Chemosphere. (2021) 274:129784. 10.1016/j.chemosphere.2021.129784 33548643

[16] Chen Y Cao S Nie J Zhao X Wang B Duan X. Food intake. In: Duan X, Zhao X, Wang B, Chen Y, Cao S, editors. Highlights of the Chinese Exposure Factors Handbook (Adults). Cambridge, MA: Academic Press (2015). p. 27–30.

[17] Cao S Zhao X Wang L Wang B Chen Y Duan X. Body weight. In: Duan X, Zhao X, Wang B, Chen Y, Cao S, editors. Highlights of the Chinese Exposure Factors Handbook (Adults). Cambridge, MA: Academic Press (2015). p. 53–4.

[18] Wang X Sato T Xing B Tao S. Health risks of heavy metals to the general public in Tianjin, China via consumption of vegetables and fish. Sci Total Environ. (2005) 350:28–37. 10.1016/j.scitotenv.2004.09.044 16227070

[19] Sun H Liu C Wang S Liu Y Liu M. Dissipation, residues, and risk assessment of spirodiclofen in citrus. Environ Monit Assess. (2013) 185:10473–7. 10.1007/s10661-013-3345-6 23880916

[20] Chien LC Hung TC Choang KY Yeh CY Meng PJ Shieh MJ. Daily intake of TBT, Cu, Zn, Cd and As for fishermen in Taiwan. Sci Total Environ. (2002) 285:177–85. 10.1016/S0048-9697(01)00916-0 11874040

[21] Crump KS. The linearized multistage model and the future of quantitative risk assessment. Hum Exp Toxicol. (1996) 15:787–98. 10.1177/096032719601501001 8906427

[22] USEPA. Drinking Water Standards and Health Advisories. In: Water O, editor. Washington, DC: U.S. Environmental Protection Agency (2018).

[23] Osborne K Crittenden A. Evolution of the Human Diet: What We Can Learn From Hunters and Gatherers. Las Vegas, NV: Center for Academic Enrichment and Outreach; University of Nevada (2013).

[24] National Health Commission PRC Ministry Ministry of Agriculture and Rural Affairs PRC State Administration for Market Regulation PRC. National food safety standard - Maximum residue limits for pesticides in food. GB (2019) 15–17.

[25] FAO. List of Pesticide Evaluated by JMPR and JMPS-Chlorothalonil. (2015). Available online at: https://www.fao.org/agriculture/crops/thematic-sitemap/theme/pests/lpe/lpe-c/en/.

[26] Jang M Moon H Kim T Yuk D Kim J Park S. Dietary risk assessment for pesticide residues of vegetables in Seoul, Korea. Korean J Food Nutr. (2010) 43:404–12. 10.4163/kjn.2010.43.4.404

[27] Jankowska M Lozowicka B Kaczyński P. Comprehensive toxicological study over 160 processing factors of pesticides in selected fruit and vegetables after water, mechanical and thermal processing treatments and their application to human health risk assessment. Sci Total Environ. (2019) 652:1156–67. 10.1016/j.scitotenv.2018.10.324 30586803

[28] Mullin CA Frazier M Frazier JL Ashcraft S Simonds R. van Engelsdorp D, et al. High levels of miticides and agrochemicals in North American apiaries: implications for Honey Bee Health. PloS ONE. (2010) 5:e9754. 10.1371/journal.pone.0009754 20333298 PMC2841636

[29] Chatzicharisis I Thomidis T Tsipouridis C Mourkidou-Papadopoulou E Vryzas Z. Residues of six pesticides in fresh peach-nectarine fruits after preharvest treatment. Phytoparasitica. (2012) 40:311–7. 10.1007/s12600-012-0231-7

[30] Minu M Roca M Hlihor RM Cozma P Gavrilescu M. Modeling of health risk associated with the intake of pesticides from Romanian fruits and vegetables. Sustainability. (2020) 12:10035. 10.3390/su122310035

[31] Mahmoud HA El-Hefny DE Helmy RMA. Environmental view for chlorothalonil on tomato and pepper fruits and soil field in Egypt: risk assessment and pre-harvest gap. Int J Environ Anal Chem. (2019) 101:639–47. 10.1080/03067319.2019.1670823

[32] Mozzachio AM Rusiecki JA Hoppin JA Mahajan R Patel R Beane-Freeman L. Chlorothalonil exposure and cancer incidence among pesticide applicator participants in the agricultural health study. Environ Res. (2008) 108:400–3. 10.1016/j.envres.2008.07.018 18801479 PMC2936501

[33] Alexander DD Weed DL Mink PJ Mitchell ME. A weight-of-evidence review of colorectal cancer in pesticide applicators: the agricultural health study and other epidemiologic studies. Int Arch Occup Environ Health. (2012) 85:715–45. 10.1007/s00420-011-0723-7 22159924

[34] Zhang D Tang J Zhang G Wu X Sun Q Jia C. Deposition, dissipation, metabolism and dietary risk assessment of chlorothalonil in open field-planted cabbage. J Food Composit Anal. (2021) 102:104008. 10.1016/j.jfca.2021.104008

[35] European Food Safety A Bellisai G Bernasconi G Brancato A Carrasco Cabrera L Ferreira L. Evaluation of confirmatory data following the Article 12 MRL review for metalaxyl-M. EFSA J. (2021) 19:6996. 10.2903/j.efsa.2021.6996 34976161 PMC8691143

