# Supplementary material for: Risk Assessment of Chlorothalonil as a Probable Human Carcinogen on Selected Vegetables in an Eastern China Province
Source: Front Public Health. 2022 Jul 6;10:917269. doi: 10.3389/fpubh.2022.917269 (PMC9298499; doi:10.3389/fpubh.2022.917269)
Supplement: Supplementary file 1 [file Data_Sheet_1.pdf]

Frontiers in Public Health

Original article: Risk Assessment of Chlorothalonil as a Probable Human Carcinogen on Selected Vegetables in an Eastern China Province

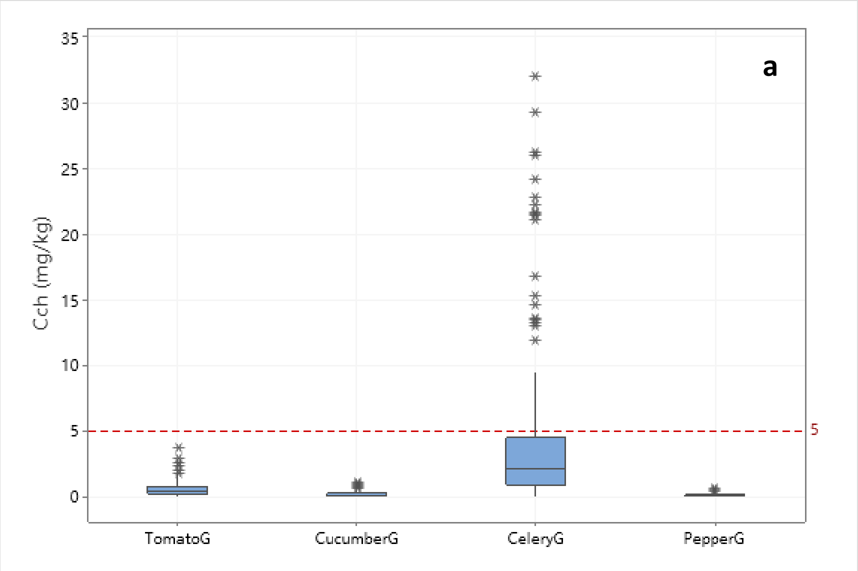

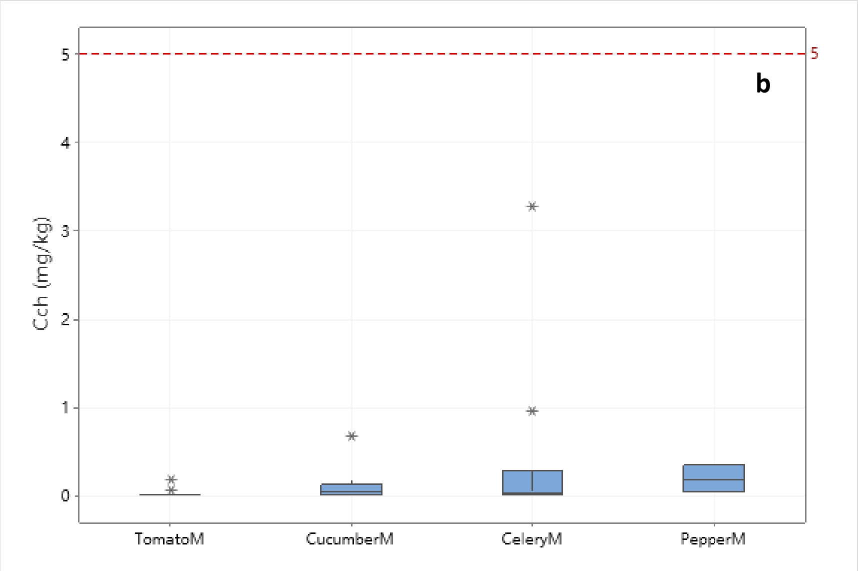

Supplementary File 1. chlorothalonil residue concentration on vegetable samples collected (a) with minimum pre-harvest interval from greenhouse and (b) from markets

Supplementary File 2. Estimated mean non-carcinogenic risk for each vegetable of different population groups

		Tomato	Cucumber	Celery	Pepper
Male Adult	Scenario1	0.0267	0.0155	0.143	0.00425
	Scenario2	0.00076	0.00580	0.00133	0.0129
	Scenario3	0.338	0.703	0.338	0.338
Female Adult	Scenario1	0.0282	0.0160	0.151	0.00449
	Scenario2	0.00080	0.00601	0.00141	0.0136
	Scenario3	0.357	0.729	0.357	0.357
Children	Scenario1	0.0255	0.0137	0.137	0.00406
	Scenario2	0.00073	0.00514	0.00127	0.0123
	Scenario3	0.323	0.623	0.323	0.323

Supplementary File 3. Estimated mean carcinogenic risk for each vegetable of each population group

		Tomato	Cucumber	Celery	Pepper
Male Adult	Scenario1	2.855E-04	1.650E-04	1.530E-03	4.543E-05
	Scenario2	8.143E-06	6.194E-05	1.422E-05	1.380E-04
	Scenario3	3.606E-03	7.479E-03	3.606E-03	3.606E-03
Female Adult	Scenario1	2.658E-04	1.509E-04	1.425E-03	4.229E-05
	Scenario2	7.580E-06	5.665E-05	1.324E-05	1.284E-04
	Scenario3	3.357E-03	6.843E-03	3.357E-03	3.357E-03
Children	Scenario1	1.281E-04	6.864E-05	6.869E-04	2.038E-05
	Scenario2	3.653E-06	2.577E-05	6.381E-06	6.191E-05
	Scenario3	1.619E-03	3.118E-03	1.619E-03	1.619E-03

*: the threshold cancer risk value: 0.0001

Supplementary File 4. Probabilities of risk exceeding 10^{-4} for each vegetable type of each population group

		Tomato	Cucumber	Celery	Pepper
Male Adult	Scenario1	82.62%	65.64%	94.59%	29.40%
	Scenario2	1.26%	38.55%	21.83%	64%
	Scenario3	/	/	/	/
Female Adult	Scenario1	81.88%	66.15%	95.52%	20.18%
	Scenario2	1.26%	36.91%	21.48%	63.28%
	Scenario3	/	/	/	/
Children	Scenario1	59.64%	38.94%	90.49%	4.80%
	Scenario2	0.15%	20.61%	16.69%	21.01%
	Scenario3	/	/	/	/
